# Epicardial adipose tissue in patients with systemic sclerosis

**DOI:** 10.1093/ehjimp/qyad037

**Published:** 2023-11-03

**Authors:** Xu Wang, Steele C Butcher, Rinchyenkhand Myagmardorj, Sophie I E Liem, Victoria Delgado, Jeroen J Bax, Jeska K De Vries-Bouwstra, Nina Ajmone Marsan

**Affiliations:** Department of Cardiology, Leiden University Medical Center, Albinusdreef 2, Leiden 2333 ZA, The Netherlands; Department of Cardiology, Beijing Anzhen Hospital, Capital Medical University, Beijing Institute of Heart Lung and Blood Vessel Disease, XCF3+6R6, Chaoyang, Beijing 100029, China; Department of Cardiology, Leiden University Medical Center, Albinusdreef 2, Leiden 2333 ZA, The Netherlands; Department of Cardiology, Royal Perth Hospital, Victoria Square, Perth WA 6000, Western Australia, Australia; Department of Cardiology, Mongolia-Japan Teaching Hospital, Mongolian National University of Medical Sciences, Botanic street, Ulaanbaatar 13270, Mongolia; Department of Rheumatology, Leiden University Medical Center, Albinusdreef 2, Leiden 2333 ZA, The Netherlands; Department of Cardiology, Leiden University Medical Center, Albinusdreef 2, Leiden 2333 ZA, The Netherlands; Hospital University Germans Trias i Pujol, Fundació Institut d’Investigació en Ciències de la Salut Germans Trias i Pujol, Carretera de Canyet, s/n, Badalona 08916, Spain; Department of Cardiology, Leiden University Medical Center, Albinusdreef 2, Leiden 2333 ZA, The Netherlands; Heart Center, University of Turku and Turku University Hospital, U-sairaala, Kiinamyllynkatu 4-8, Turku 20521, Finland; Department of Cardiology, Mongolia-Japan Teaching Hospital, Mongolian National University of Medical Sciences, Botanic street, Ulaanbaatar 13270, Mongolia; Department of Cardiology, Leiden University Medical Center, Albinusdreef 2, Leiden 2333 ZA, The Netherlands

**Keywords:** systemic sclerosis, epicardial adipose tissue, left ventricular diastolic dysfunction, mortality

## Abstract

**Aims:**

Epicardial adipose tissue (EAT) has emerged as a mediator between systemic inflammatory disorders and cardiovascular disease, and may therefore play a role in the pathophysiology of cardiac involvement in systemic sclerosis (SSc). The aim of this study was to assess the correlation between EAT and left ventricular (LV) function, and to determine the prognostic value of EAT in patients with SSc.

**Methods and results:**

Consecutive patients with SSc who underwent non-contrast thorax computed tomography and echocardiography were included. EAT mass was quantified using dedicated software. The study endpoint was all-cause mortality. A total of 230 SSc patients (age 53 ± 15 years, 14% male) were included. The median value of EAT mass was 67 g (interquartile range: 45–101 g). Patients with increased EAT mass (≥67 g) showed more impaired LV diastolic function as compared with patients with less EAT mass (<67 g), and even after adjusting for age and comorbidities, EAT mass was independently associated with LV diastolic function parameters. During a median follow-up of 8 years, 42 deaths occurred. Kaplan–Meier analysis showed that patients with increased EAT mass had higher all-cause mortality rate as compared with patients with less EAT mass (29% vs. 7%; *P* < 0.001). In the multivariable analysis, EAT was independently associated with all-cause mortality after adjusting for important covariates (HR: 1.006; 95% CI: 1.001–1.010).

**Conclusion:**

In patients with SSc, EAT is independently associated with LV diastolic dysfunction and higher mortality rate.

## Graphical Abstract

**Graphical abstract:**
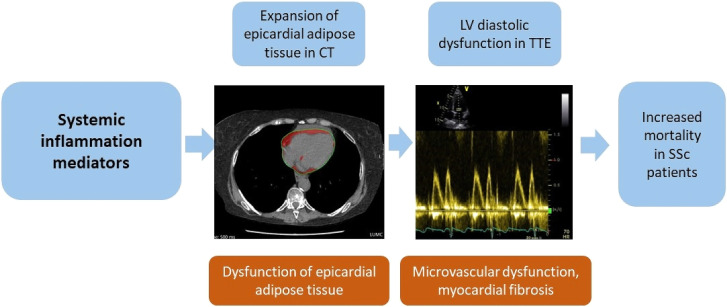

## Introduction

Systemic sclerosis (SSc) is an autoimmune connective tissue disease characterized by microvascular injury and progressive fibrosis of skin and visceral organs.^1^ The heart is frequently involved in SSc, with cardiovascular abnormalities being one of the major causes of mortality in these patients.^2^ Cardiac manifestations typically include myocarditis, heart failure with systolic and/or diastolic dysfunction, pulmonary arterial hypertension, pericardial involvement, and arrhythmias.^3^ Particularly, previous studies indicated that left ventricular (LV) diastolic dysfunction is relatively common in patients with SSc, ranging from 18% to 62%,^4,5^ and is an important predictor of mortality.^6^ Potential substrates leading to LV diastolic dysfunction are the presence of diffuse and focal myocardial fibrosis, possibly secondary to inflammation or to ischaemia from microcirculation impairment.^7–9^ Also, accumulation of epicardial adipose tissue (EAT) has been associated with local inflammation that may involve the adjacent cardiac tissues, causing microvascular dysfunction and fibrosis.^10–12^ When the process adjoins the LV, it impairs myocardial distensibility, increasing LV stiffness and filling pressures and leading to LV diastolic dysfunction.^8,13^ There is already growing evidence that EAT is related to cardiovascular risk factors, coronary artery disease, atrial fibrillation, and heart failure with preserved ejection fraction (HFpEF).^14–16^ We therefore hypothesized that EAT extent, as assessed by computed tomography (CT), may be related to cardiac involvement in patients with SSc and particularly to LV diastolic dysfunction. Aims of the present study were two-fold: (i) to explore the association between EAT and cardiac dysfunction in patients with SSc; and (ii) to determine the prognostic value of EAT in these patients.

## Methods

### Study population

Consecutive SSc patients referred to Leiden University Medical Center (Leiden, The Netherlands) and enrolled in the Leiden Comprehensive Care in Systemic Sclerosis (CCISS) Cohort (REU 043/SH/sh) were included in this study from April 2009 to May 2015.^17,18^ Patients were diagnosed according to the American College of Rheumatology/the European League Against Rheumatism classification criteria for SSc.^19^ Multidisciplinary team care and organ screening including thorax CT and echocardiography were performed to assess interstitial lung disease (ILD) and cardiac involvement for all patients, who gave written informed consent for this assessment as according to the approval of the Leiden University Medical Center Medic Ethical Committee. However, only patients who underwent echocardiography within one month from the initial thorax CT were enrolled. In addition, patients with missing baseline clinical data and uninterpretable thorax CT images were excluded. Also, patients with significant valvular heart disease and pericardial involvement were excluded to avoid interference with the EAT measures.

Disease-related characteristics included among others the modified Rodnan skin score (mRSS), SSc subtype (limited or diffuse cutaneous), the time since diagnosis, and the time since Raynaud. Complete laboratory testing was performed, including renal function, N-terminal pro-brain natriuretic peptide (NT-proBNP), C-reactive protein (CRP), and creatine phosphokinase (CPK). Pulmonary function was assessed by performing spirometry tests according to the American Thoracic Society/European Respiratory Society recommendations,^20,21^ and included maximum O_2_ uptake during exercise (VO2max) and diffusion capacity of the lung for carbon monoxide (DLCO). Pulmonary hypertension (PAH) was diagnosed according to 2015 ESC/ERS guidelines for the diagnosis and treatment of pulmonary hypertension.^22^

### Echocardiography

Transthoracic echocardiography was performed using a commercially available ultrasound system equipped with 3.5 MHz transducers (Vivid 7 and E9, GE-Vingmed, Horten, Norway). Standard M-mode and two-dimensional, colour, pulsed, and continuous wave Doppler images were acquired. The images were digitally stored and retrospectively measured (EchoPAC Version 203.0.1, GE Medical Systems, Horten, Norway). LV volumes were measured on the apical four- and two-chamber views and LV ejection fraction (LVEF) was calculated using the Simpson biplane method.^23^ The left atrial volume was measured on the apical four- and two-chamber views according to the method of discs and indexed to body surface area [left atrial volume index (LAVI)]. LV internal diameters, interventricular septal thickness (IVST), and posterior wall thickness (PWT) were measured at end diastole (and end systole for LV diameter). LV mass was calculated based on the recommended formula and indexed to body surface area [left ventricular mass index (LVMI)].^23^ The peak velocity of the tricuspid regurgitation (TR) jet was derived from continuous wave Doppler. Inspiratory collapsibility and the inferior vena cava diameter were measured to determine right atrial pressure and calculate pulmonary pressure. Pulsed wave Doppler of mitral inflow was used to measure the peak early diastolic (E) wave and late diastolic (A) wave. The ratio of peak early diastolic wave-to-late diastolic wave was calculated. The septal and lateral peak early diastolic mitral annular velocities (Eʹ) were measured using tissue Doppler imaging in the apical four-chamber view. Subsequently, the ratio between E and Eʹ was calculated to assess LV filling pressures.

### CT acquisition

Non-contrast thorax CT scans were acquired using a 64-detector row helical scanner (Aquilion 64, Toshiba Medical Systems, Toshiba Medical Systems, Otawara, Japan) or a 320-detector row volumetric scanner (Aquilion ONE, Toshiba Medical Systems, Otawara, Japan). The CT datasets were reconstructed at a slice thickness of either 2.5 or 3.0 mm in hybrid iterative reconstruction technique (iDose5, Philips Healthcare, Best, The Netherlands).

### Quantification of EAT mass

**Figure 1:**
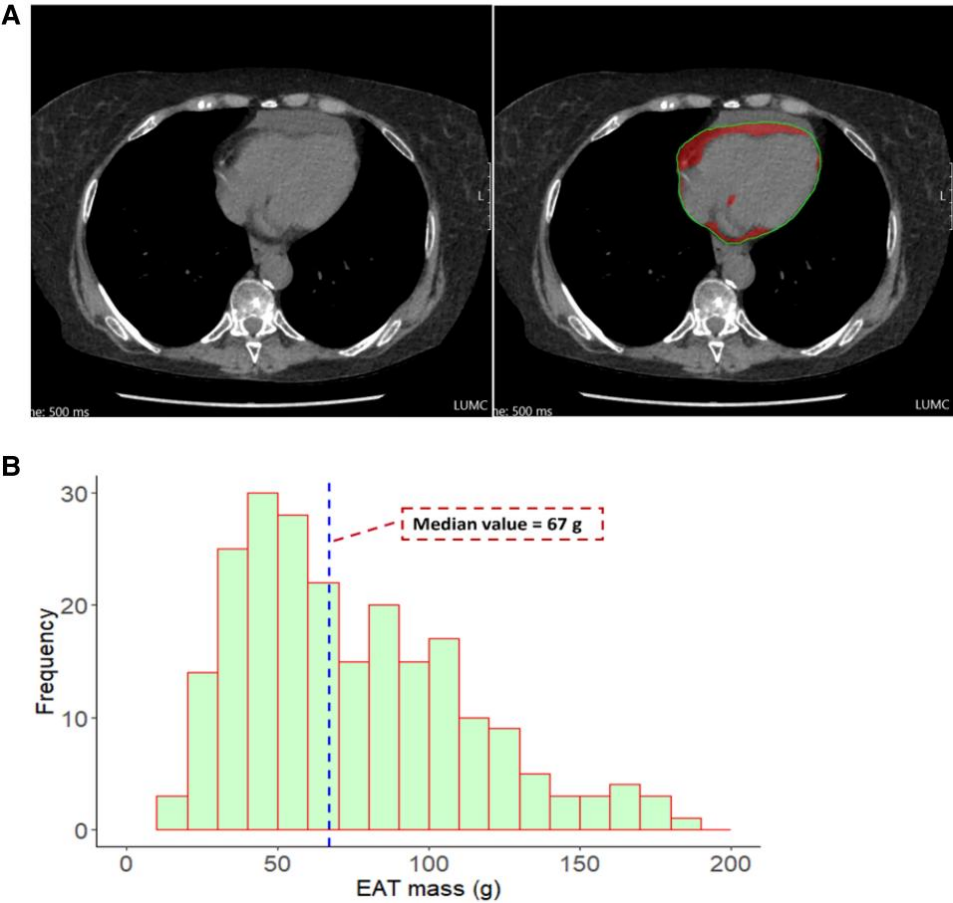
EAT mass assessment. (*A*) Example of quantification of EAT mass. Manual tracing was performed from the pulmonary artery bifurcation (superior limit) to the PDA (inferior limit), and the adipose tissue within the pericardium was automatically recognized by the software as tissue with Hounsfield Units (HU) between −195 and −45. (*B*) Histograms of frequencies of EAT mass. Median value of EAT mass in study cohort was 67 g. EAT, epicardial adipose tissue; PDA, posterior descending coronary artery.

EAT was measured in gram (mass) using the MASS software (Leiden University Medical Center, Leiden, The Netherlands) as described before.^24^ Briefly, a cardiac cross-sectional view of thorax non-contrast CT was acquired from the reconstructed four-chamber views with 2 mm slice thickness. The limits of the heart were defined as the pulmonary artery bifurcation (superior limit) to the posterior descending coronary artery (inferior limit). The pericardium was manually traced from the pulmonary artery bifurcation until the posterior descending coronary artery. The EAT was automatically identified by the software as tissue with HU between −195 and −45 within the region of pericardium^24^ (*Figure 1A*). To define the intra- and inter-observer variability, the EAT mass measurements were repeated for 10 randomly selected patients > 4 weeks apart by the same observer (X.W. blinded to the echocardiographic results) on the same thorax CT images and by a second independent observer (R.M.).

### Follow-up and study endpoint

The study endpoint was all-cause mortality. Medical records review and survival data were obtained from the hospital information systems (EPD-Vision and EZIS; Leiden University Medical Center, Leiden, The Netherlands) or the Social Security Death Index. Follow-up began from the date that CT was performed.

### Statistical analysis

Normally distributed continuous variables were presented as mean ± standard deviation; and non-normally distributed continuous variables were presented as median values and interquartile range (IQR). The Student’s *t*-test was used for comparison of normally distributed continuous variables, while the Mann–Whitney *U* test was used for non-normally distributed continuous variables. Categorical data were presented as frequencies with percentages and were compared using the χ^2^ test. For the reproducibility of EAT measurements, the intra-class correlation coefficient (ICC) was calculated to assess the inter- and intra-observer variability. Correlation between EAT and echocardiographic cardiac functional parameters was evaluated in multivariable linear regression analysis. The cumulative survival rate was calculated using Kaplan–Meier analysis, and comparison between groups was performed using the log-rank test. Cox regression analyses were used to investigate the association between clinical, echocardiographic, and CT variables and all-cause mortality. Variables with a *P*-value < 0.05 at the univariable analysis were included in the Cox proportional hazards backward stepwise model. The hazard ratio (HR) and 95% confidence interval were reported. The incremental value of EAT over baseline clinical variables for the risk of all-cause mortality was assessed using the likelihood ratio test. All statistical tests were two-sided, and a *P*-value < 0.05 was considered statistically significant. Statistical analysis was performed using SPSS version 25.0 (IBM Corporation, Armonk, NY, USA) and R version 4.1.1 (R Foundation for Statistical Computing, Vienna, Austria).

## Results

### Patient characteristics

A total of 230 patients [mean age 53 ± 15 years and 33 (14%) males] with SSc were enrolled. In the overall population, the median value of EAT mass was 67 g (IQR: 45 to 101 g) (*Figure 1B*), by which the subjects were therefore dichotomized. Other baseline characteristics of the study cohort are summarized in *Table 1*. Patients with increased EAT (≥67 g) were older as compared with patients with less EAT (<67 g), had more frequently PAH, and had also significantly higher prevalence of comorbidities including diabetes and hypertension. Moreover, patients with increased EAT had significantly higher values of CRP and NT-proBNP, lower mean estimated glomerular filtration rate (eGFR), and more impaired DLCO as compared with patients with less EAT. Finally, cardiovascular medications were more frequently used in patients with increased EAT, including angiotensin-converting enzyme inhibitors or angiotensin II receptor blockers, calcium channel blockers, beta-blockers, and diuretics. These results suggest that patients with higher EAT had also more progressed disease stage (including cardiac, renal, and pulmonary involvement) and more comorbidities, with a worse cardiovascular burden (diabetes and hypertension).

**Table 1:** Baseline clinical characteristics of the total SSc population and divided based on the median value of EAT mass

	Total population	EAT ≥ 67 g	EAT < 67 g	*P*-value
	(*n* = 230)	(*n* = 115)	(*n* = 115)	
Clinical variables				
Age, y	53 ± 15	60 ± 11	45 ± 14	<0.001
Male, *n* (%)	33(14.3)	24(20.9)	9(7.8)	0.005
BMI, kg/m^2^	24.6 ± 4.3	26.0 ± 4.4	23.1 ± 3.6	<0.001
Diffuse SSc, *n* (%)	51(23.1)	24(21.6)	27(24.5)	0.458
HTN, *n* (%)	58(25.2)	39(33.9)	19(16.5)	0.002
DM, *n* (%)	11(4.8)	9(7.8)	2(1.7)	0.031
CAD, *n* (%)	8(3.5)	6(5.2)	1(0.8)	0.150
AF, *n* (%)	7(3.2)	6(5.5)	1(0.9)	0.055
History of smoking, *n* (%)	110(47.8)	61(53.0)	49(42.6)	0.113
mRSS	5 ± 8	6 ± 9	4 ± 5	0.124
ILD, *n* (%)	91(41.2)	52(46.8)	39(35.5)	0.085
PAH, *n* (%)	8(3.6)	7(6.3)	1(0.9)	0.032
Time since Raynaud, y	13 ± 14	15 ± 15	11 ± 12	0.013
Time since diagnosis, y	5 ± 7	6 ± 8	4 ± 6	0.014
Laboratory findings				
eGFR, mL/min/1.73 m^2^	88(72–103)	82(69–96)	95(79–106)	<0.001
NT-proBNP, ng/L	95.8(53.6–170.5)	107.6(59.2–252.4)	79.0(49.1–137.6)	0.005
CRP, mg/L	0(0–5)	0(0–6)	0(0–0)	0.002
CPK, U/L	83(61–118)	93(60–134)	79(61–109)	0.239
ANA-positive, *n* (%)	211(95.5)	105(94.6)	106(96.4)	0.423
ACA-positive, *n* (%)	87(39.4)	44(39.6)	43(39.1)	0.933
ATA-positive, *n* (%)	54(24.4)	23(20.7)	31(28.3)	0.197
DLCO%	64.7 ± 19.1	59.5 ± 18.5	70.0 ± 18.2	<0.001
VO2max%	87.0 ± 23.7	85.1 ± 22.4	88.9 ± 24.9	0.262
Immunosuppressive medication, *n* (%)				
Corticosteroids	31(13.5)	18(15.7)	13(11.3)	0.334
Cyclophosphamide	3(1.3)	2(1.7)	1(0.9)	0.561
Methotrexate	29(12.6)	18(15.7)	11(9.6)	0.164
Azathioprine	11(4.8)	9(7.8)	2(1.7)	0.031
Cardiovascular medications, *n* (%)				
ACEI/ARB	85(37.0)	57(49.6)	28(24.3)	<0.001
CCB	108(47.0)	64(55.7)	44(38.3)	0.008
Beta-blockers	21(9.1)	17(14.8)	4(3.5)	0.003
Diuretics	30(13.0)	22(19.1)	8(7.0)	0.006

Values are mean ± standard deviation if normally distributed and median (interquartile range) if not normally distributed.

ACA, anti-centromere antibodies; ACEI, angiotensin-converting enzyme inhibitor; AF, atrial fibrillation; ANA, anti-nuclear antibodies; ARB, angiotensin II receptor blocker; ATA, anti-topoisomerase antibodies; BMI, body mass index; CAD, coronary artery disease; CCB, calcium channel blocker; CPK, creatine kinase; CRP, C-reactive protein; DLCO, diffusion capacity of the lung for carbon monoxide; DM, diabetes mellitus; eGFR, estimated glomerular filtration rate; HTN, hypertension; ILD, interstitial lung disease; MRSS, modified Rodnan skin score; NT-proBNP, N-terminal pro-brain natriuretic peptide; PAH, pulmonary artery hypertension; VO2max, maximum O_2_ uptake during exercise.

### Echocardiography

The echocardiographic variables of the study population are displayed in *Table 2*. Patients with increased EAT had significantly larger IVST and PWT as compared with those with less EAT. Furthermore, patients with increase EAT had larger LAVI, larger LVMI, reduced mean Eʹ, increased E/Eʹ, and higher TR peak velocity compared with those with less EAT. No significant differences in LV end-diastolic volume, LV end-systolic volume, and LVEF were observed between the two groups.

**Table 2:** Baseline echocardiographic and CT characteristics in the total population and divided based on the median value of EAT

	Total population	EAT ≥ 67 g	EAT < 67 g	*P*-value
	(*n* = 230)	(*n* = 115)	(*n* = 115)	
Echocardiographic parameters				
LVEDV, mL	83.5 ± 24.3	82.3 ± 26.2	84.7 ± 22.7	0.562
LVESV, mL	32.6 ± 13.6	32.2 ± 15.0	32.9 ± 12.2	0.694
LVEF, %	61.7 ± 6.8	61.7 ± 7.0	61.6 ± 7.0	0.921
LVMI, g/m^2^	148.2 ± 42.3	156.4 ± 39.6	140.0 ± 43.4	0.003
IVST, mm	8.9 ± 1.8	9.2 ± 1.7	8.7 ± 1.9	0.048
PWT, mm	8.9 ± 1.6	9.1 ± 1.6	8.7 ± 1.7	0.037
LAVI, mL/m^2^	23.9 ± 9.4	27.0 ± 9.9	20.8 ± 7.9	<0.001
E/A	1.1 ± 0.4	1.0 ± 0.3	1.3 ± 0.4	<0.001
Eʹ, cm/s	9.0 ± 2.9	7.5 ± 2.2	10.5 ± 2.8	<0.001
E/E′	9.5 ± 3.9	10.8 ± 4.3	8.2 ± 2.8	<0.001
TRV, m/s	2.4 ± 0.4	2.5 ± 0.4	2.3 ± 0.3	<0.001
CT parameter				
EAT, g	67 (45–101)	100 (82–122)	45 (35–56)	<0.001

Values are mean ± standard deviation if normally distributed and median (interquartile range) if abnormally distributed.

CT, computed tomography; E, peak early diastolic mitral flow velocity; E′, peak early diastolic mitral annular tissue velocity; EAT, epicardial adipose tissue; IVST, interventricular septum thickness; LAVI, left atrial volume index; LV, left ventricular; LVEDV, left ventricle end-diastolic volume; LVEF, left ventricular ejection fraction; LVESV, left ventricular end-systolic volume; LVMI, left ventricular mass index; PWT, posterior LV wall thickness; TRV, tricuspid regurgitation velocity.

### Association between EAT and cardiac function

Univariable linear regression analyses showed that EAT was significantly associated to LV diastolic function parameters including LAVI, LVMI, Eʹ, E/Eʹ, and TR peak velocity, while there was no significant association between EAT and LVEF (see Supplementary data online, *Table S1*).

At the multivariable linear regression analyses, EAT showed an independent association with LV diastolic function parameters including LAVI (*B* = 0.031, *P* = 0.025), LVMI (*B* = 0.139, *P* = 0.036), E/E′ (*B* = 0.025, *P* < 0.001), and Eʹ (*B* = −0.012, *P* < 0.001) after correction for clinical characteristics including age, sex, hypertension, diabetes, and eGFR, indicating that increased EAT is associated with impaired LV diastolic function (*Table 3*).

**Table 3:** Multivariable linear regression analysis for EAT and echocardiographic parameters of LV diastolic function (therefore corrected for demographics and comorbidities)

	LAVI		LVMI		E/E′		Eʹ		TRV	
	*B* coeff	*P*-value	*B* coeff	*P*-value	*B* coeff	*P*-value	*B* coeff	*P*-value	*B* coeff	*P*-value
Age	0.261	<0.001	0.359	0.101	0.079	<0.001	−0.111	<0.001	0.158	<0.001
Male	0.691	0.681	28.139	<0.001	−0.780	0.222	−0.028	0.944	0.851	0.615
DM	0.271	0.923	9.068	0.919	0.995	0.327	−0.705	0.291	0.085	0.974
HTN	−2.908	0.034	−0.388	0.952	0.532	0.303	−0.273	0.412	−2.262	0.075
eGFR	0.014	0.572	0.105	0.001	−0.001	0.921	0.007	0.247	−0.017	0.461
EAT	0.031	0.025	0.139	0.036	0.025	<0.001	−0.012	<0.001	0.025	0.112

DM, diabetes mellitus; E, peak early diastolic mitral flow velocity; E′, peak early diastolic mitral annular tissue velocity; EAT, epicardial adipose tissue; eGFR, estimated glomerular filtration rate; HTN, hypertension; LAVI, left atrial volume index; LVMI, left ventricular mass index; TRV, tricuspid regurgitation velocity.

### EAT and outcome

**Figure 2:**
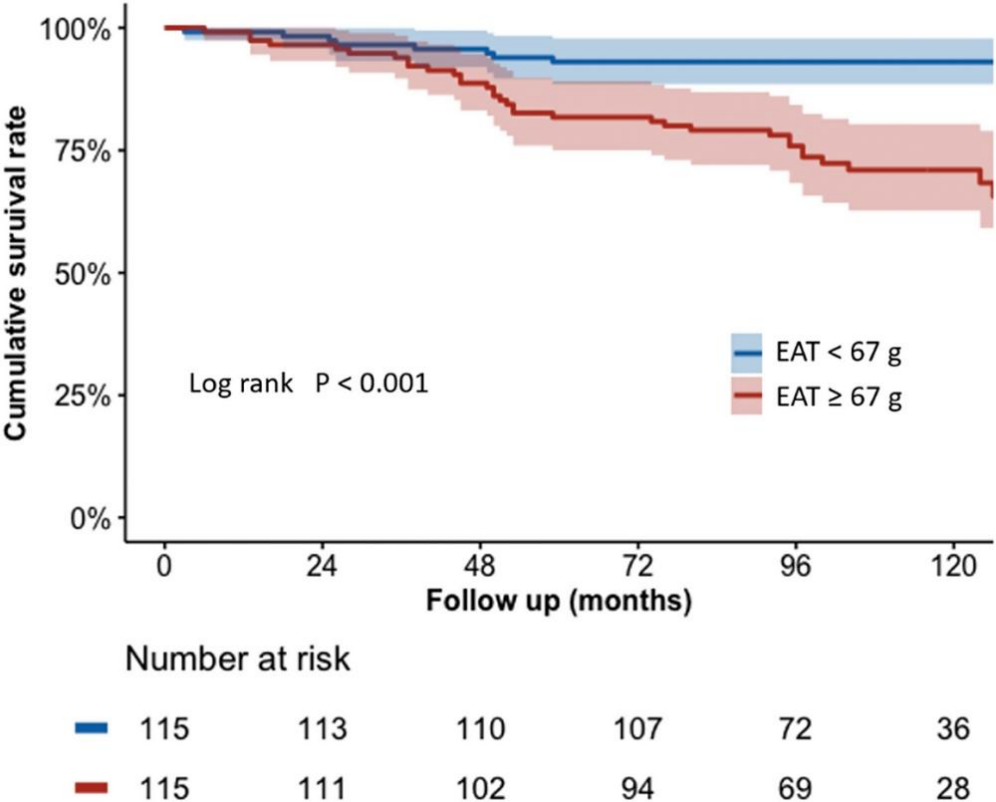
Kaplan–Meier curves for all-mortality. Time to death according to baseline EAT < 67 g (less) and EAT ≥ 67 g (more). The lighter shaded colour around the curves indicates a 95% confidence interval. EAT, epicardial adipose tissue.

During a median follow-up of 8 years (IQR 7–10 years), 42 patients (18%) died. Survival analysis demonstrated that patients with increased EAT (≥67 g) experienced significantly higher rates of all-cause mortality (29.6% vs. 7.0%; *P* < 0.001) (*Figure 2*), compared with patients with less EAT (<67 g).

Univariable cox regression analyses demonstrated significant associations between all-cause mortality and age, ILD, DLCO, CRP, NT-proBNP, and EAT mass (*Table 4*). Because of the total number of events, five variables including age, DLCO (preferred over ILD to represent pulmonary involvement), NT-proBNP, CRP, and EAT mass were ultimately included in the multivariable Cox proportional hazards backward stepwise model. In this analysis, age, DLCO, and EAT mass were independently associated with all-cause mortality (*Table 4*). Of note, when considering a combined endpoint of all-cause mortality, heart failure hospitalization, myocardial infarction, and arrhythmias, at the multivariable analysis EAT remained also independently associated with the endpoint [HR 1.005 (1.001–1.009), *P* = 0.008] together with NT-proBNP and DLCO (data not shown in the tables).

**Table 4:** Univariable and multivariable analyses to evaluate the association between EAT and all-cause mortality

Variable	Univariable analysis		Multivariable analysis	
	HR (95% CI)	*P*-value	HR 95% CI	*P*-value
Age (per one year increase)	1.072 (1.044–1.101)	<0.001	1.047 (1.017–1.079)	0.002
Male (yes/no)	1.877 (0.922–3.821)	0.082		
Diffuse SSc (yes/no)	1.785 (0.913–3.489)	0.090		
PAH (yes/no)	1.436 (0.990–2.084)	0.056		
ILD (yes/no)	3.738 (1.901–7.331)	<0.001		
Time since Raynaud (per one year increase)	1.001 (0.979–1.023)	0.918		
Autoantibodies				
0	Reference			
1	0.949 (0.251–3.578)	0.938		
2	0.556 (0.153–2.023)	0.373		
3	1.379 (0.411–4.629)	0.603		
CRP (per one unit increase)	1.046 (1.024–1.069)	<0.001	NS	
eGFR (per one unit increase)	0.990 (0.978–1.001)	0.087		
DLCO (per one unit increase)	0.975 (0.968–0.981)	<0.001	0.978 (0.971–0.985)	<0.001
LVEF (per one unit increase)	0.976 (0.937–1.017)	0.249		
NT-proBNP (per one unit increase)	1.121 (1.083–10160)	<0.001	NS	
EAT (per gram increase)	1.009 (1.006–1.012)	<0.001	1.006 (1.001–1.010)	0.010

Autoantibodies is a categorical variable (0 = ANA−, 1 = ATA+, 2 = ACA+, and 3 = other).

ACA, anti-centromere antibodies; ANA, anti-nuclear antibodies; ATA, anti-topoisomerase antibodies; DLCO, diffusion capacity of the lung for carbon monoxide; DM, diabetes mellitus; EAT, epicardial adipose tissue; E′, peak early diastolic mitral annular tissue velocity; eGFR, estimated glomerular filtration rate; HTN, hypertension; ILD, interstitial lung disease; LVEF, left ventricular ejection fraction; NT-proBNP, N-terminal pro-brain natriuretic peptide; PAH, pulmonary artery hypertension; SSc, systemic sclerosis.

### Incremental prognostic value of EAT

The addition of EAT to a Cox model for all-cause mortality that included age and DLCO resulted in a significantly improved model fit as assessed by using the likelihood ratio test (*P* = 0.019).

### Reproducibility

Measurement of EAT mass in 10 randomly selected patients showed excellent intra- and inter-observer reproducibility. The inter-observer ICC was 0.993 (95% CI 0.815–0.999), and the intra-observer ICC was 0.997 (95% CI 0.963–0.999) for EAT measurement.

## Discussion

The present study examined a large cohort of SSc patients, exploring for the first time the association between EAT, LV function, and clinical outcomes. The extent of EAT measured by non-contrast CT was significantly and independently associated with LV diastolic dysfunction and with higher mortality rates.

### Association between EAT, severity of SSc, and LV diastolic dysfunction

The current study showed that patients with increased EAT had higher levels of CRP, longer time to diagnosis (or to Raynaud), higher levels of NT-proBNP, lower eGFR values, and DLCO, suggesting an association between EAT and severity of SSc and further organ damage.

Previous studies showed that several systemic inflammatory diseases, including rheumatoid arthritis, systemic lupus erythematosus, and psoriasis, are accompanied by an increase in EAT mass, which is proportional to the duration and severity of the underlying disease.^25–27^ In SSc, increased EAT volume was also suggested to be associated with the presence and severity of the disease.^28^ In particular, the activation of aldosterone, leptin, and neprilysin observed in systemic inflammatory disease may facilitate the accumulation and dysfunction of EAT.^8^ In addition to systemic inflammation, numerous metabolic disorders (such as diabetes, insulin resistance, and metabolic syndrome) are accompanied by the expansion and inflammation of visceral adipose tissue^29–32^; therefore, assessment of the EAT extent should take into account also the presence of these comorbidities.

On the other hand, because of its endocrine and paracrine activity, secreting pro-inflammatory and anti-inflammatory cytokines and chemokines, EAT may play a role in the pathophysiology of cardiac damage. Previous studies have suggested EAT to be related to coronary atherosclerosis, microcirculatory dysfunction, myocardial fibrosis, and LV hypertrophy,^33–39^ and therefore to be involved in the development of coronary artery disease, atrial fibrillation, and most importantly HFpEF.^14–16^ This study shows that EAT is associated with echocardiographic parameters of LV diastolic function including LAVI, LVMI, Eʹ, and E/Eʹ, and independently of other risk factors and comorbidities. It therefore supports the hypothesis that EAT plays a role in the cardiovascular involvement in SSc, and mainly in the development of LV diastolic dysfunction (overt LV systolic dysfunction was not observed in this cohort).

### EAT and prognosis

Many chronic systemic inflammatory disorders are accompanied by an increased risk of heart failure, particularly HFpEF,^24,40–43^ and this risk is independent of macrovascular coronary heart disease. Particularly in SSc, presence of LV diastolic dysfunction has been shown to be of independent prognostic value.^6^ As above-mentioned, EAT, by playing a role in the development of myocardial fibrosis, LV hypertrophy, and impaired distensibility, may be therefore an important marker of cardiac involvement. In addition, EAT may also help general risk stratification of SSc patients by reflecting the severity of the disease and of multiorgan involvement. To the best of our knowledge, this study is the first demonstrating an association between EAT and clinical outcome in a large SSc cohort, importantly after correction for clinical characteristics and LV function. Considering that most patients with SSc undergo a non-contrast thorax CT, EAT measure could be easily implemented in the routine assessment of these patients and have impact in their management. Importantly, EAT could also represent a novel therapeutic target: because of its rapid metabolism and potential for modification, targeting EAT with drugs such as glucagon-like peptide 1 and sodium-glucose cotransporter-2 inhibitor agonists may reshape the landscape of pharmacotherapy, providing cardiovascular protection beyond just SSc management.

### Study limitations

This study has all of the inherent limitations associated with a single centre, retrospective study design. However, the systematic and standardized evaluation of all the patients, according to a prospective cohort study design, supports the robustness of the findings; of note, only patients with the CT scan and the echocardiography performed at the same visit were included and patients with inadequate CT quality were excluded, which may have introduced a selection bias. Finally, a direct demonstration of the EAT inflammatory activity could not be provided neither by imaging or laboratory tests. The role that EAT plays in pathogenesis of cardiac involvement in SSc requires therefore future investigation.

### Conclusion

In patients with SSc, EAT is associated with LV diastolic dysfunction and higher mortality rate, independently of traditional risk factors. The mechanisms underlying this association warranted to be explored.

## Supplementary Material

qyad037_Supplementary_Data

## Data Availability

The data underlying this article will be shared on reasonable request to the corresponding author.

## Lead author biography

**Nina Ajmone Marsan, MD, PhD, FESC**

**Assistant Professor, senior cardiologist, expert in valvular heart disease and cardiovascular imaging, Leiden University Medical Center, The Netherlands**

Dr Nina Ajmone Marsan is a senior cardiologist, specializing in non-invasive cardiovascular imaging and currently working at the Leiden University Medical Center (LUMC, The Netherlands), where she is Assistant Professor and supervises several Dutch and international PhD students. Her main research topic is the clinical applications of imaging in cardiovascular disease with special focus on valvular heart disease (particularly for patients referred for trans-catheter interventions), cardiomyopathies, and systemic disease. She is also the principal investigator of translational research projects in mitral and aortic valve disease and the coordinator of the LUMC Heart Valve Center. She is in the board of EACVI as Vice president elect 2022–2024 and as Chair of the Task Forces and Shared Interest Groups (2022–2024). She is Associate Editor of the European Heart Journal of the European Heart Journal Cardiovascular Imaging of the Journal of the American Society of Echocardiography. She is in the Congress Program Committee of the ESC 2023 and course director of the EACVI TOE teaching course, of the PCR Madrid Imaging Valve and of the ‘3D echocardiography: meet the experts’ course.
