# Supplementary material for: Epicardial adipose tissue in patients with systemic sclerosis
Source: Eur Heart J Imaging Methods Pract. 2023 Nov 3;1(2):qyad037. doi: 10.1093/ehjimp/qyad037 (PMC11195713; doi:10.1093/ehjimp/qyad037)
Supplement: qyad037_Supplementary_Data [file qyad037_Supplementary_Data.docx]

**Supplementary table 1. Univariable linear regression analysis for EAT and echocardiographic parameters of cardiac function.**

|  | **EAT (g)** | |
| --- | --- | --- |
|  | **B (95% CI)** | **P-value** |
| **LV systolic function parameter** |  |  |
| LVEF | -0.001 (-0.020 to 0.017) | 0.882 |
| **LV diastolic function parameters** |  |  |
| E’ | -0.030 (-0.037 to -0.023) | <0.001 |
| E/E’ | 0.037 (0.028 to 0.046) | <0.001 |
| LAVI, ml/m2 | 0.059 (0.035 to 0.084) | <0.001 |
| LVMI, g/m2 | 0.216 (0.104 to 0.329) | <0.001 |
| TRV, m/s | 0.002 (0.001 to 0.004) | <0.001 |

E, peak early diastolic mitral flow velocity; E′, peak early diastolic mitral annular tissue velocity; EAT, epicardial adipose tissue; LAVI, left atrial volume index; LV, left ventricular; LVMI, left ventricular mass index; TRV, tricuspid regurgitation velocity.
